# Intestinal epithelial Notch-1 protects from colorectal mucinous adenocarcinoma

**DOI:** 10.18632/oncotarget.26086

**Published:** 2018-09-11

**Authors:** David Dunkin, Alina C. Iuga, Sanda Mimouna, Carolyn L. Harris, Jean-Vianney Haure-Mirande, Dominique Bozec, Garabet Yeretssian, Stephanie Dahan

**Affiliations:** ^1^ Department of Pediatric Gastroenterology, Icahn School of Medicine at Mount Sinai, New York, NY 10029, USA; ^2^ The Mindich Child Health and Development Institute, Icahn School of Medicine at Mount Sinai, New York, NY 10029, USA; ^3^ Department of Pathology and Cell Biology, Columbia University Medical Center, New York, NY 10032, USA; ^4^ The Precision Immunology Institute, Icahn School of Medicine at Mount Sinai, New York, NY 10029, USA; ^5^ Immunology and Autoimmunity Research Department, Hospital for Special Surgery Research Institute, New York, NY 10021, USA; ^6^ Division of Clinical Immunology, Department of Medicine, Icahn School of Medicine at Mount Sinai, New York, NY 10029, USA; ^7^ Division of Rheumatology, Department of Medicine, Icahn School of Medicine at Mount Sinai, New York, NY 10029, USA; ^8^ Department of Neurology, Icahn School of Medicine at Mount Sinai, New York, NY 10029, USA; ^9^ Brain Tumor Nanotechnology Laboratory, Department of Neurosurgery, Icahn School of Medicine at Mount Sinai, New York, NY 10029, USA; ^10^ Tisch Cancer Institute, Icahn School of Medicine at Mount Sinai, New York, NY 10029, USA; ^11^ The Leona M. and Harry B. Helmsley Charitable Trust, New York, NY 10169, USA; ^12^ Sobi, Inc. Waltham, MA 02452, USA

**Keywords:** Notch-1, mucinous adenocarcinoma, colorectal cancer

## Abstract

Increasing evidence links Notch-1 signaling with the maintenance of intestinal architecture and homeostasis. Dysfunction in the common Notch-1 pathway transcription factor recombinant binding protein suppressor of hairless (RBP-J) is associated with loss of epithelial barrier integrity and aberrant conversion of proliferative crypt cells into goblet cells. Furthermore, we have recently discovered that epithelial Notch-1 is indispensable in bridging innate and adaptive immunity in the gut and is required for supporting protective epithelial pro-inflammatory responses. Yet, the epithelial specific function of Notch-1 in intestinal tumorigenesis remains unknown. We generated *Villin-Cre/Notch-1*^*fl/fl*^ (*VN*^*-/-*^) mice that are selectively deficient in Notch-1 in intestinal epithelial cells. Intestinal epithelial Notch-1 preserved barrier function and integrity, whereas lack of epithelial Notch-1 induced goblet cell hyperplasia, spontaneous serrated lesions, multifocal low- and high-grade dysplasia and colonic mucinous neoplasms in mice. Over time, *VN*^*-/-*^ mice displayed high occurrence of colorectal mucinous adenocarcinomas, which correlated with increased levels of mitogenic, angiogenic and pro-tumorigenic gene expression. Finally, we found that the expression of Notch-1 is significantly reduced in human colorectal mucinous adenocarcinoma when compared to colorectal adenocarcinoma. Taken together, our findings reveal a novel and critical protective role for Notch-1 in controlling intestinal tumorigenesis.

## INTRODUCTION

The mucosal surface of the intestinal tract is covered with a single layer of epithelium that constitutes a physical and immunological barrier against a variety of foreign antigens derived from food, pathogens and commensal bacteria. Intestinal stem cells located at the crypt bottom sustain continuous turnover of the epithelium by giving rise to transit-amplifying cells that undergo proliferation and differentiation into absorptive (enterocytes) and secretory (Paneth, goblet and enteroendocrine cells) cell lineages [1, 2]. Keeping a balanced cellular composition of the intestinal epithelium is essential to maintain homeostasis and protect from various gastrointestinal disorders. While the mechanisms controlling the intestinal epithelial cell fate are not completely known, multiple signaling pathways (e.g. Notch, Wnt, Hedgehog, etc.) have been involved in dictating and influencing progenitor cell proliferation and differentiation [3–6].

The Notch signaling pathway plays a key role in the embryonic and adult gut epithelium, and is required both for maintenance of intestinal stem cells and for a proper balance of differentiation between secretory and absorptive cell lineages [7, 8]. In the absence of Notch signaling, the epithelium is remodeled and stem cells preferentially produce secretory cells with reciprocal decline in absorptive cells. Blocking Notch signaling with γ-secretase inhibitors or by deletion of the Notch effector recombinant binding protein suppressor of hairless (RBP-J) induces loss of proliferating stem and progenitor cells, and a global secretory cell hyperplasia in the intestine of mice [9, 10]. Notch promotes the absorptive fate through upregulation of the transcriptional repressor hairy and enhancer of split-1 (Hes-1), which, in turn inhibits the expression of atonal homolog-1 (Atoh-1), a master transcription factor for all secretory cells [11]. Conditional deletion of Atoh-1 in the intestinal epithelium rescues the RBP-J phenotype in mice further indicating that Notch signaling negatively controls secretory commitment through repression of Atoh-1 [11, 12]. Conversely, transgenic expression of a constitutively active form of Notch-1, the intracellular domain of Notch (NICD), in the intestinal epithelium affects the amplification of immature progenitor cells at the expense of secretory cells [13, 14].

Increasing evidence underscores the importance of Notch signaling in intestinal tumorigenesis, yet unlike in acute T-cell leukemia, activating mutations in Notch receptors are unusual in colorectal cancer [15, 16]. Conflicting reports have highlighted the role of the Notch pathway in tumorogenesis in the intestine. However, the directionality of the Notch pathway dysregulation in tumorigenesis remains unclear. The goal of our study is to decipher the role of epithelial Notch-1 in colorectal tumor formation, development and progression.

In this study, we show that the *Villin-Cre/Notch-1*^*fl/fl*^ (*VN*^*-/-*^) mice, which have a deletion of Notch-1 under the villin promoter in the intestinal epithelial cells, develop serrated lesions, multifocal epithelial dysplasia and spontaneous colorectal mucinous neoplasms in a background of colonic mucosa with impaired barrier integrity, and enhanced goblet cell metaplasia. The incidence of colorectal mucinous adenocarcinomas in *VN*^*-/-*^ mice correlated with increased levels of mitogenic, angiogenic and pro-tumorigenic genes. Concomitantly, Notch-1 expression is significantly reduced in human colorectal mucinous adenocarcinoma compared to non-mucinous colorectal adenocarcinoma. Our findings emphasize the implication of epithelial Notch-1 in i) controlling intestinal tumorigenesis, and, ii) highlighting a molecular signature in a subset of patients with colorectal mucinous adenocarcinoma that may benefit from targeted screening and subsequent therapeutics.

## RESULTS

### Notch-1 depletion in intestinal epithelial cells induces barrier dysfunction

We have previously reported that Notch-1 controls intestinal barrier function at homeostasis, both *in vitro* and *in vivo* [17–19]. Moreover, the intestinal epithelial depletion of RBP-J, a transcription factor that mediates signaling downstream of Notch receptors, leads to epithelial barrier dysfunction and subsequent development of spontaneous chronic colitis [20]. In order to deepen our knowledge of the role of epithelial Notch-1, we generated a Notch-1 conditional knockout mouse strain under the villin promoter (*Villin-Cre;Notch-1*^*fl/fl*^; termed *VN*^*-/-*^). These mice were born at the expected Mendelian ratio, and deletion of Notch-1 expression within the intestinal epithelial compartment was confirmed by qPCR (data not shown). To delineate the *in vivo* implication of Notch-1 in intestinal epithelial integrity, colonic permeability, resistance and tight junction protein expression were assessed in WT and *VN*^*-/-*^ mice at 10 weeks of age.

**Figure 1:**
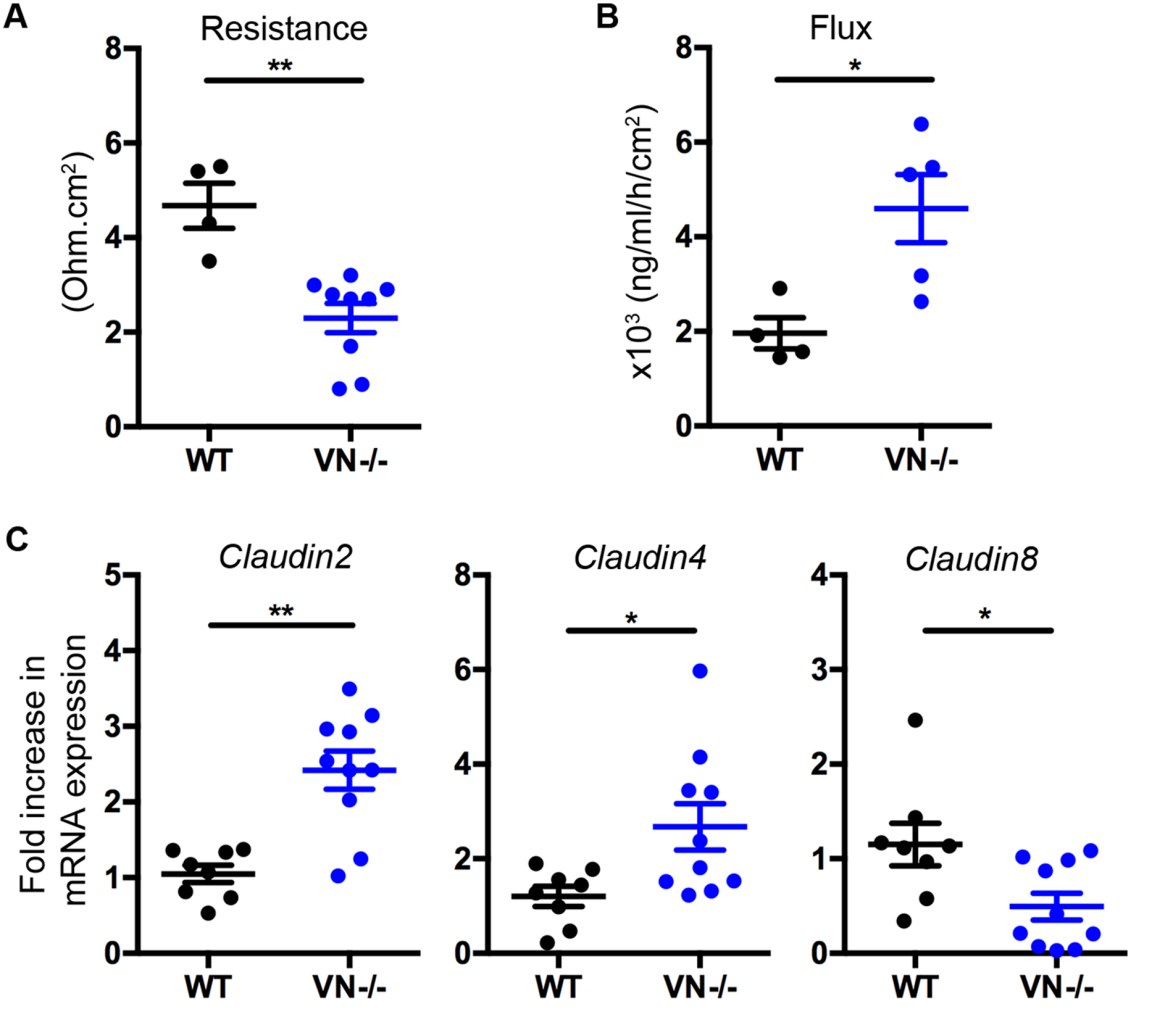
Epithelial Notch-1 maintains intestinal barrier function and homeostasis **(A)** Changes in epithelial resistance as a measure of passive transcellular and paracellular ion transport, and **(B)** alterations in paracellular permeability for small molecules were evaluated. Distal colon was harvested from wild-type C57BL/6J (WT) and *VN*^*-/-*^ mice at 10 weeks of age, and transepithelial resistance was evaluated along with paracellular permeability by measuring FITC-dextran flux from the mucosal to the serosal compartment using spectrofluorometry. **(C)** *Claudin-2*, *-4*, and *-8* mRNA expression was assessed in the distal colon of WT (n=8) and *VN*^*-/-*^ (n=10) mice at 10 weeks of age by quantitative PCR (qPCR). Data represent the mean ± SEM of at least 4 mice per group. ^*^p<0.05, ^**^p<0.01 (Mann Whitney two-tailed test).

Transepithelial resistance of the colon tissue of *VN*^*-/-*^ mice was significantly decreased and was concomitant with an increase in FITC-dextran flux across the mucosa when compared to WT mice (Figure 1A and 1B), suggesting an impaired intestinal barrier integrity in the absence of epithelial Notch-1. These findings were accompanied by increased claudin-2 and -4 and decreased claudin-8 mRNA expression in the distal colon of *VN*^*-/-*^ mice (Figure 1C), pointing toward a role for Notch-1 in maintaining a homeostatic tight junction protein stoichiometry.

Altogether, these results underscore the importance of epithelial Notch-1 in maintaining barrier integrity and intestinal homeostasis.

### Lack of intestinal epithelial Notch-1 promotes secretory cell hyperplasia

**Figure 2:**
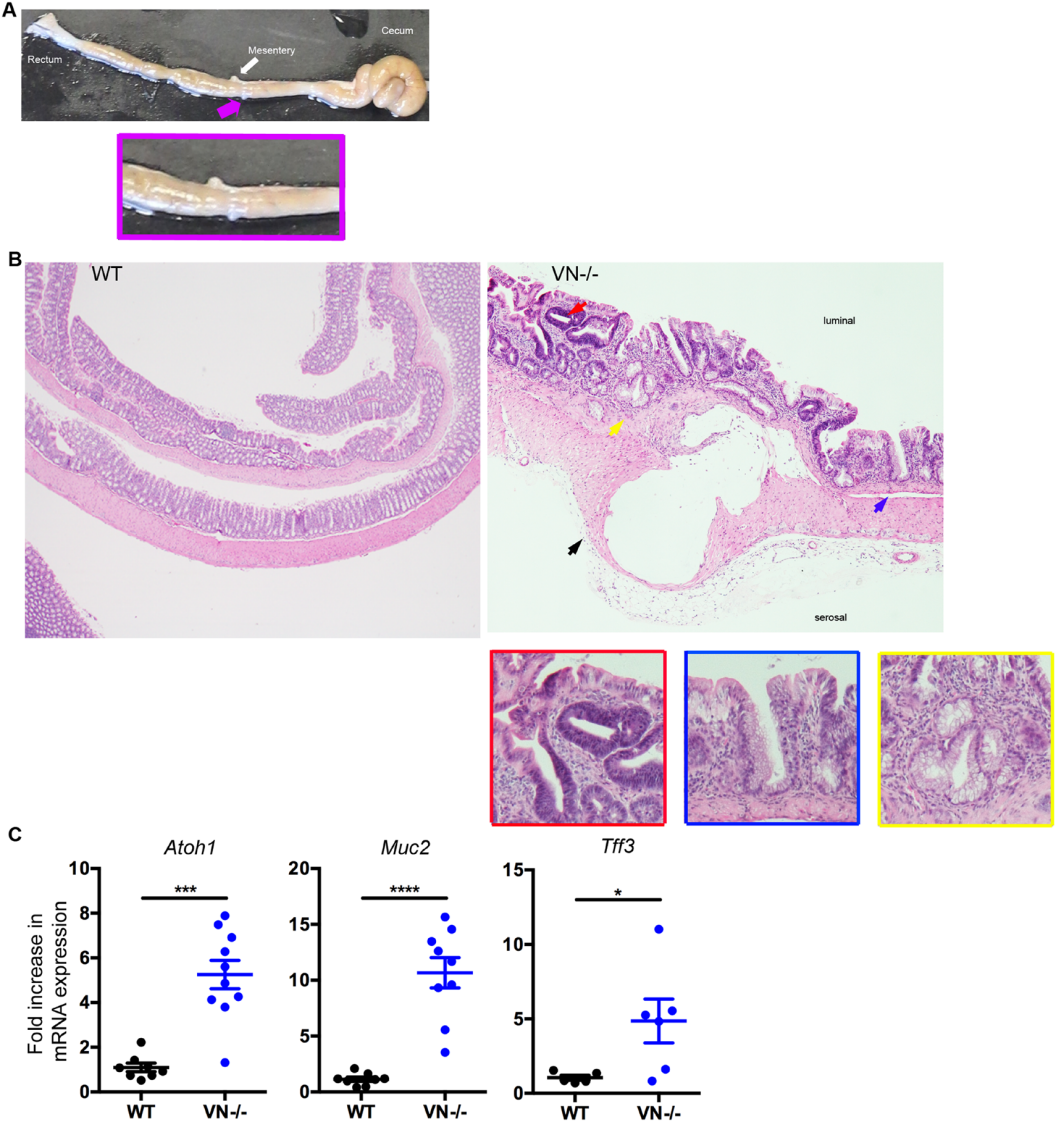
*VN*^*-/-*^ mice are highly susceptible to spontaneous colorectal mucinous adenocarcinoma **(A)** Macroscopic picture of the colon from a 10-week old *VN*^*-/-*^ mouse demonstrating a mucinous lesion grossly: pink arrow and inset. **(B)** Hematoxylin and eosin (H&E, original magnification ×4) staining of colon tissue section from 10-week old WT and *VN*^*-/-*^ mice. The red arrow indicates low-grade dysplasia; the yellow arrow represents Goblet cell hyperplasia; the blue arrow designates serrated lesions; and the black arrow denotes a mucinous lesion with epithelial lining. Magnified views of the marked colored arrows are shown on the right panels. **(C)** Fold induction of *Atoh-1*, *Muc2*, and *Tff3* gene expression in the distal colon of WT (n=5-8) and *VN*^*-/-*^ (n=6-10) mice. Data represent the mean ± SEM of at least 5 mice per group. ^*^p<0.05, ^***^p<0.001, ^****^p<0.0001 (Mann Whitney two-tailed test).

Notch-1 signaling affects intestinal homeostasis by tightly controlling the differentiation pathway of the enterocyte lineage and promoting the absorptive cell fate. Repression of epithelial Notch-1 signaling through deletion of the Notch-1 effector RBP-J or blocking its activity by γ-secretase inhibition has been reported to shift differentiation into the secretory pathway, with goblet cells being the default result of this shift [9, 10]. To test the implication of intestinal epithelial Notch-1 in the balance between absorptive and secretory lineage differentiation specifically in the colon, we used the *VN*^*-/-*^ mice and characterized these mice for signs of excessive secretory cell differentiation. Macroscopically, cystic like lesions are apparent in the colon (Figure 2A, pink arrow and inset). Histological examination of the colons of *VN*^*-/-*^ mice at 10 weeks of age showed diffuse goblet cell hyperplasia and associated architectural changes consisting of basal gland “boot-shaped” dilatation, crypt branching and glandular distortion (Figure 2B, blue and yellow arrows and inset), features that have been described in human sessile serrated polyps [21] and are markedly different from the appearance of normal WT colon.

To further investigate the role of Notch-1 in epithelial lineage fate, we assessed the mRNA expression of goblet cell markers by qPCR. *Atoh-1*, mucin-2 (*Muc2*) and trefoil factor-3 (*Tff3*) mRNA levels were all significantly up-regulated in the *VN*^*-/-*^ mice compared to WT mice (Figure 2C), confirming that lack of epithelial Notch-1 induces excessive goblet cell hyperplasia in the colon.

Taken together, our data show that Notch-1 is essential in maintaining a homeostatic epithelial lineage differentiation.

### Notch-1-deficient mice develop spontaneous colorectal mucinous adenocarcinoma

In addition to diffuse goblet cell hyperplasia with associated glandular architectural changes similar to those described in serrated colonic polyps, the *VN*^*-/-*^ mice at 10 weeks of age showed multiple foci of low-grade dysplasia, characterized by scattered clusters of glands with epithelial proliferation, nuclear pseudostratification, nuclear enlargement and hyperchromasia, increased nuclear to cytoplasmic ratio, increased mitotic activity, but preserved nuclear polarity (Figure 2A, red arrow and inset). Focally, high-grade dysplasia was also present in the colons of these mice, featuring marked nuclear atypia, loss of nuclear polarity and complex, cribriform glandular architecture. Adjacent to foci of dysplasia, submucosal mucinous lesions were identified in most of the *VN*^*-/-*^ mice. These lesions consisted of well-delineated cysts filled with mucin (Figure 2A, black arrow). The larger lesions displayed epithelial lining and detached intracystic epithelial cells, with mild to moderate epithelial atypia. These mucinous lesions were unifocal and located in the proximal colon which is analogous to the ascending colon in humans. None of the *VN*^*-/-*^ mice analyzed at 10 weeks of age showed any signs of peritoneal or liver implants (data not shown). No evidence of pericystic or lamina propria inflammation or cyst rupture was seen in association with these mucinous lesions, ruling out a herniation or colitis-associated cystica profunda (Figure 2A).

Mucinous adenocarcinomas, commonly seen on the right side of the colon have been associated with the serrated pathway to colorectal carcinogenesis in human pathology [22]. In this context, taking into account the presence of histological changes recapitulating the human serrated neoplasia pathway, the cystic mucinous epithelial lesions seem to be consistent with a neoplastic process.

### Chronic Notch-1 deficiency in intestinal epithelial cells worsens spontaneous intestinal pathology and causes high grade dysplasia

**Figure 3:**
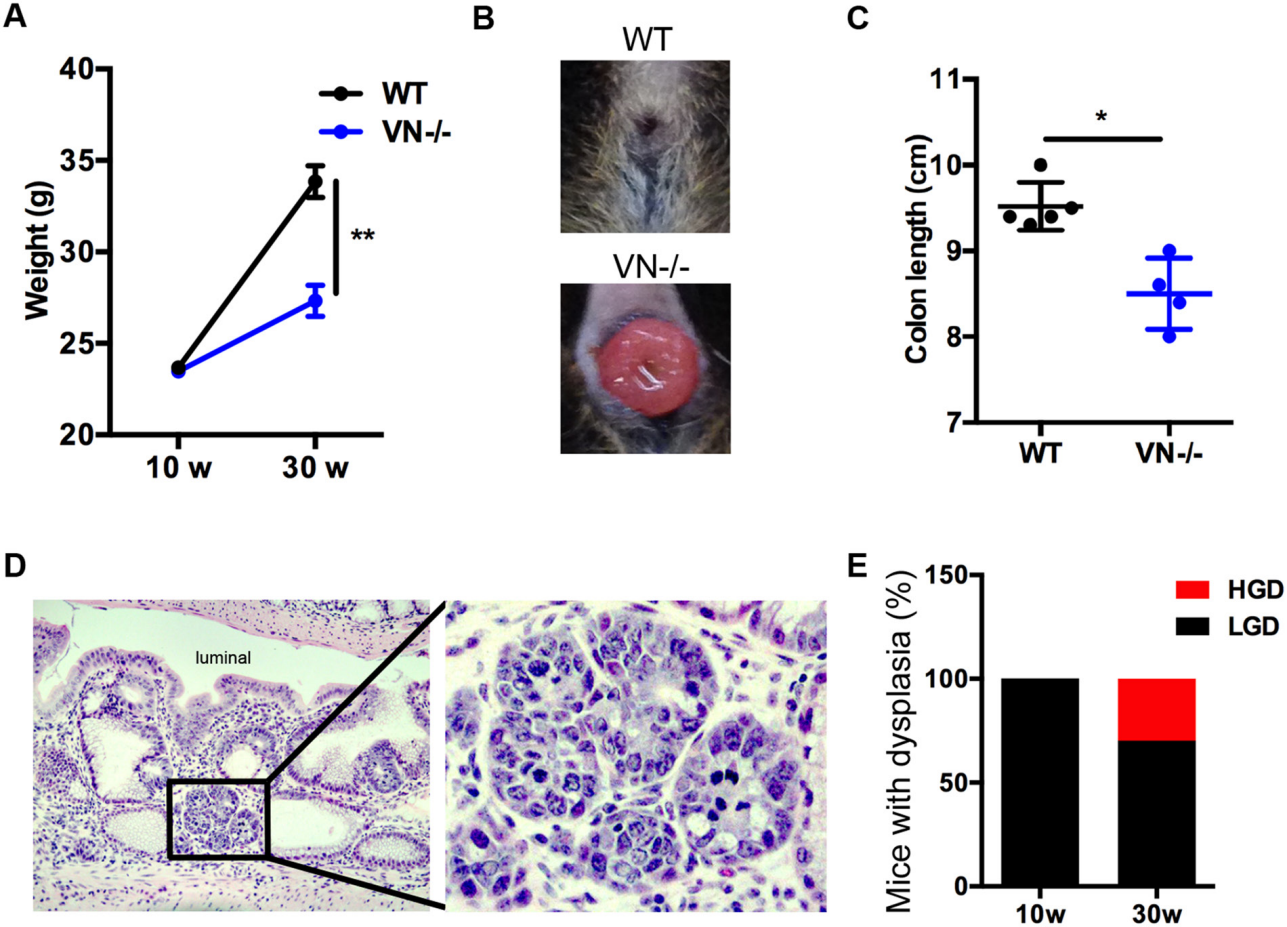
Chronic Notch-1 deficiency in intestinal epithelial cells worsens spontaneous intestinal pathology and causes high grade dysplasia **(A)** Body weight curve of WT (n=5) and *VN*^*-/-*^ (n=8) mice at 10 and 30 weeks (w) of age. **(B)** Representative image of rectal prolapse in a 30-week old *VN*^*-/-*^ mouse. **(C)** Colon length of the indicated mice at 30 weeks of age. **(D)** H&E (original magnification ×20) staining of colon tissue section from a 30-week old *VN*^*-/-*^ mouse. Focus of high-grade dysplasia (HGD; black inset) showing nuclear enlargement, rounding and hyperchromasia, and exhibiting increased nuclear to cytoplasmic ration and brisk mitotic activity. Magnified view of the marked black area is shown in the right panel. **(E)** Percentage of mice with dysplasia (HGD, high-grade dysplasia; LGD, low-grade dysplasia). Data represent the mean ± SEM of at least 4 mice per group. ^*^p<0.05, ^**^p<0.01 (Mann Whitney two-tailed test).

The spontaneous phenotype of the *VN*^*-/-*^ mice at 10 weeks of age led us to follow these mice longitudinally and investigate the putative role of epithelial Notch-1 in eliciting tumorigenesis. Mice were aged up to 30 weeks. *VN*^*-/-*^ mice gained significantly less body weight when compared to age matched WT mice (Figure 3A). This finding was coupled with the appearance of rectal prolapse in 28.2% of aging mice (Figure 3B). When mice were sacrificed, colon lengths were measured and significantly reduced in the aged *VN*^*-/-*^ mouse group (Figure 3C), indicating that the absence of epithelial Notch-1 prompts worse intestinal pathology. Histological analysis demonstrated that 30-week old *VN*^*-/-*^ mice were prone to develop colonic high-grade dysplasia (Figure 3D). While 100% of 10-week old *VN*^*-/-*^ mice showed low-grade dysplasia, 30% of 30-week old *VN*^*-/-*^ mice developed high-grade dysplasia (Figure 3E) in addition to the low-grade dysplasia, suggesting that loss of epithelial cell-intrinsic Notch-1 promotes tumorigenesis.

### Absence of Notch-1 enhances tumorigenic transformation of intestinal epithelial cells and tumor invasion

**Figure 4:**
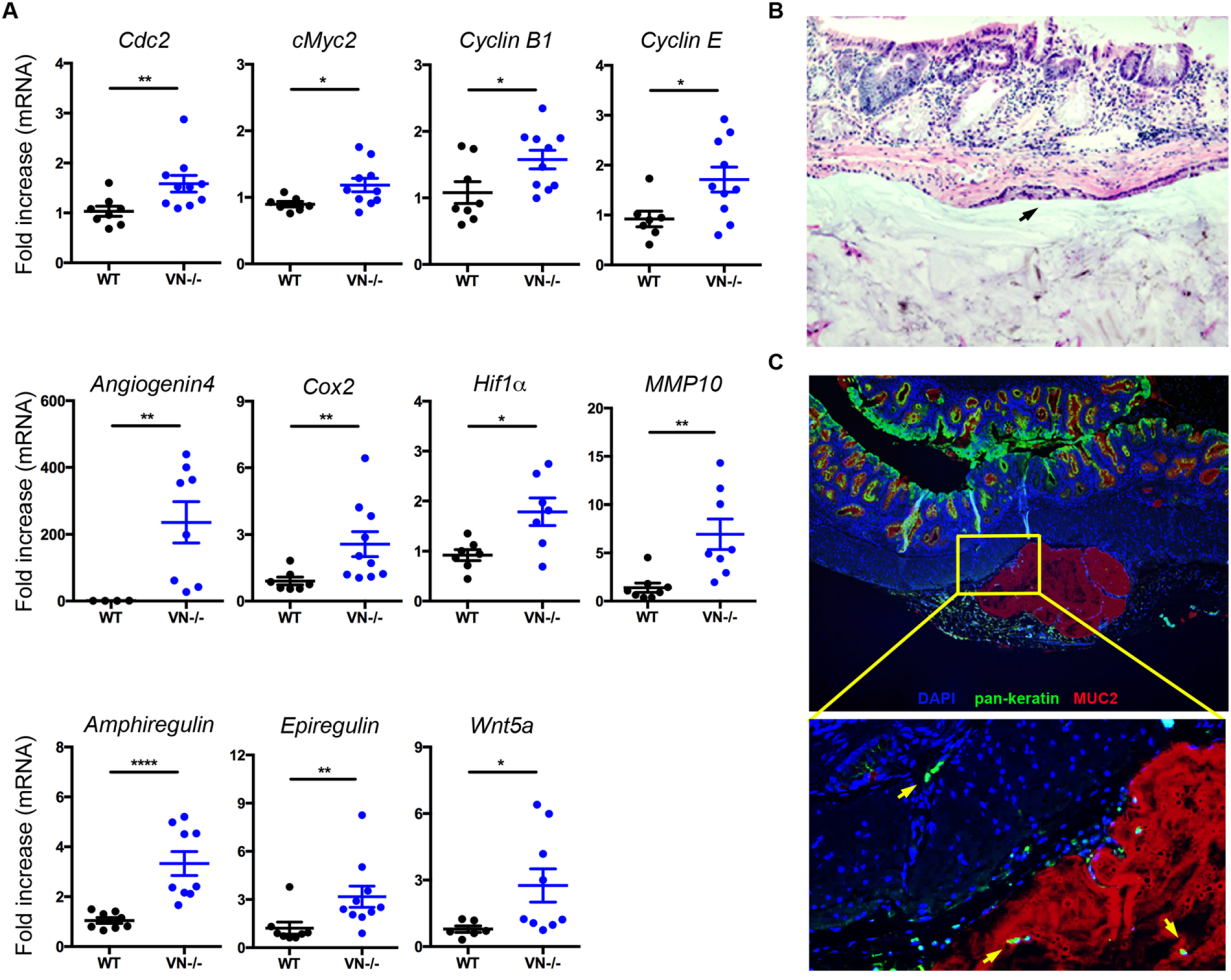
Notch-1 controls intestinal expression of pro-tumorigenic factors and tumor invasion **(A)** Fold induction in mRNA expression of angiogenic, mitogenic, and pro-tumorigenic genes in colon tissues from WT (n=6-8) and *VN*^*-/-*^ (n=7-10) mice at 30 weeks of age. **(B)** Representative section (H&E, original magnification ×20) of a mucinous neoplasm, showing a sub-mucosal epithelial-lined cyst (black arrow) filled with mucin. **(C)** Immunofluorescence staining of MUC2 in colon tissue section of a *VN*^*-/-*^ mouse at 30 weeks of age shows MUC2^+^ mucin filled cysts (red). Intestinal epithelial cells stained with Pan-Keratin (green) and nuclei with DAPI (blue). Magnified view of the marked yellow area is shown in the lower panel. Yellow arrows indicate invasive intestinal epithelial cells. Data represent mean ± SEM of at least 6 mice per genotype. ^*^p<0.05, ^**^p<0.01, ^****^p<0.0001 (Mann Whitney two-tailed test).

High-grade dysplasia is usually coupled with dysregulation in tumorigenic signals. To determine the role of epithelial Notch-1 in controlling tumor-promoting factors, we analyzed the mRNA expression of various cell cycle, mitogenic, angiogenic, and tumor-promoting genes and identified a significant increased expression of the following genes in the colonic tissues of *VN*^*-/-*^ mice compared to WT mice: cell division cycle 2 (*Cdc2*), c-Myc*2, cyclin B1*, *cyclin E, Angiogenin-4*, cyclooxygenase 2 (*Cox2*), hypoxia-inducible factor 1 alpha (*Hif1a*), matrix metalloproteinase 10 (*Mmp10*), *Amphiregulin*, *Epiregulin, and Wnt5a* (Figure 4A).

To further examine whether lack of Notch-1 confers invasive properties to the intestinal epithelial cells, colonic tissue from *VN*^*-/-*^ mice were analyzed for mucin expression and dissociation of epithelial cells as a prerequisite for tumor invasion. Histological analysis and immunofluorescent microscopy targeting MUC2 revealed aberrant accumulation of mucus in epithelial-lined submucosal cysts (Figure 4B and 4C). Notably, many detached epithelial structures were located in the colonic sub-mucosa of *VN*^*-/-*^ mice and in the MUC2^+^ cysts (Figure 4C, yellow arrows), suggesting that Notch-1-deficient epithelial cells have acquired motility and tumor invasion properties.

Collectively, these findings underscore the important role of intestinal epithelial Notch-1 in determining dissemination of tumor cells and invasion.

### Patients with mucinous colorectal adenocarcinoma exhibit decreased Notch-1 mRNA expression

**Figure 5:**
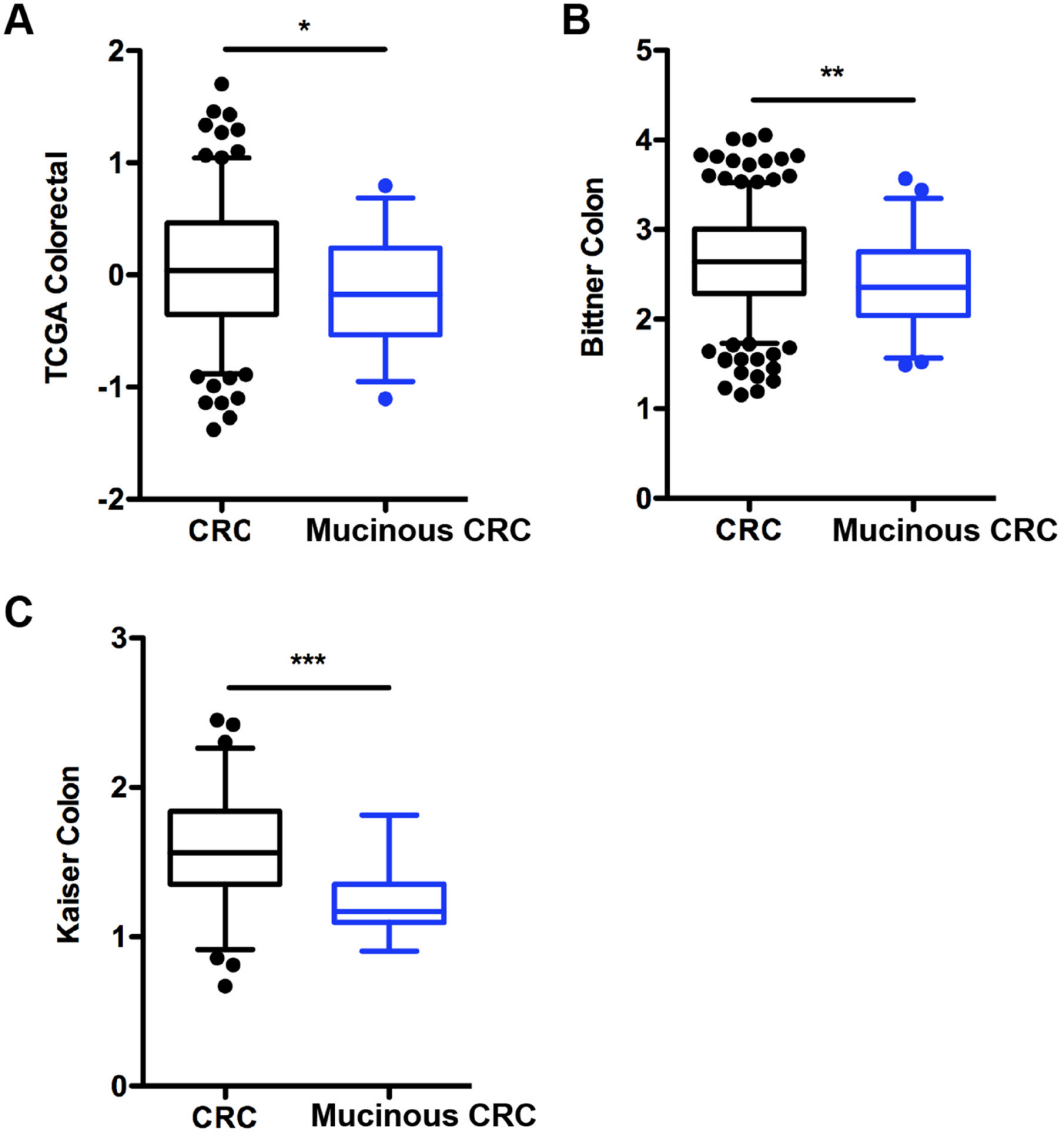
Notch-1 expression is downregulated in human colorectal mucinous adenocarcinoma *Notch-1* mRNA expression in colorectal cancer (CRC) compared with mucinous CRC tissues in **(A)** TCGA Colorectal, **(B)** Bittner colon and **(C)** Kaiser colon gene expression microarray datasets accessed using the Oncomine database (https://www.oncomine.com/). Box-and-whiskers plots depict the distribution of *Notch-1* expression within each group presented as Log2 median-centered ratios and created using GraphPad Prism 5. The whiskers are drawn down to the 5^th^ percentile and up to the 95^th^. Points below and above the whiskers are drawn as individual dots. n, CRC/Mucinous CRC=186/28; n, Bittner Colon =324/45; and n, Kaiser Colon = 75/17. ^*^p<0.05, ^**^p<0.01, ^***^p<0.001 (Mann Whitney two-tailed test).

Given our findings in the *VN*^*-/-*^ mice consistent with loss of Notch-1 promoting mucinous CRC, we examined the role of Notch-1 in human mucinous colorectal cancer (CRC). Expression of *Notch-1* mRNA was analyzed in CRC and mucinous CRC tissues in three gene expression datasets using the Oncomine^®^ database. We found a significantly reduced expression of Notch-1 mRNA in mucinous CRC when compared with CRC tissues in the TCGA Colorectal (n, CRC/Mucinous CRC=186/28); Bittner Colon (n, 324/45); and Kaiser Colon (n, 75/17) datasets (Figure 5A-5C).

These results indicate that downregulation of Notch-1 is a common feature of patients with mucinous CRC and that gene analysis should take into consideration patient stratification based on specific CRC subsets.

## DISCUSSION

Colorectal cancer is the second leading cause of cancer death in the United States [23]. The majority of colon neoplastic lesions arise through the classical adenoma-carcinoma pathway associated with KRAS mutations and chromosomal instability. However, up to one third of colon adenocarcinomas develop through the serrated pathway, classified based on the histological appearance of the crypts in the precursor polyps. Sessile serrated adenomas have been shown to be associated with colonic mucinous colloid-type adenocarcinoma [22, 24, 25]. These lesions are often overlooked and incompletely resected during screening colonoscopy and therefore only uncovered after the development of CRC and metastasis [22]. The mechanism of progression from sessile serrated adenoma to invasive carcinoma are unclear. Our *VN*^*-/-*^ murine model, where there is deletion of Notch-1 in intestinal epithelial cells, recapitulates the histological changes seen in human serrated adenomas and therefore gives us further insight into this neoplasia pathway. We report here a new molecular signature highlighting the role of epithelial Notch-1 in developing mucinous CRC in both mice and humans, emphasizing the clinical relevance of our model.

Notch-1 was found to play an important role in numerous neoplasms [26]. Its function as a tumor activator or suppressor remains controversial, appears to be cell-type and cancer-type specific [27–30].

In murine models, there is conflicting data on the role of Notch-1 signaling in CRC. Activation of Notch in APC^+/1638N^ mutant mice, which spontaneously develop intestinal adenomas, was associated with higher tumor burden and worse survival [6]. On the other hand, mutations that downregulate expression of Hes-1 or Jagged-1 (one of the Notch ligands) in APC^Min^mice reduced the proliferation of tumor cells [29, 31]. In addition, activation of Notch-1 signaling in APC^Min^ mice converted high-grade adenomas into low-grade adenomas along with suppression of about 40% of tumor-associated genes, particularly genes involved in Wnt signaling [32]. Our data demonstrates a tumor suppressive role of Notch-1. *VN*^*-/-*^ mice, which lack Notch-1 specifically in the intestinal epithelium, developed spontaneous mucinous colorectal tumors associated with adenoma/low-grade dysplasia, high-grade dysplasia, goblet cell hyperplasia and serrated changes. We further demonstrate that tumor incidence in *VN*^*-/-*^ mice correlated with increased expression in the colon of mitogenic, angiogenic and pro-tumorigenic factors.

Decreases in Notch-1 expression are known to lead to defects in the mucosal barrier and IEC differentiation and function [17], but the mechanism of how this leads to the development of dysplasia is not fully understood. The defective barrier plays an important role in dysplasia; many reports have linked defects in barrier function to bacteria-induced inflammation and to subsequent development of dysplasia, neoplasm and colorectal cancer [33–38]. Notch-1 decreases in expression lead to increases in ATOH-1 and goblet cell differentiation as well and this may directly or indirectly be linked to the development of dysplasia [39].

Mouse models of concomitant colonic serrated polyps and mucinous neoplasia have been lacking so far. Transgenic mice with intestinal expression of the EGFR ligand heparin-binding EGF-like growth factor (HB-EGF) in the intestine develop colonic serrated polyps, but no dysplasia [40]. Mice expressing Grem1 cDNA under the control of the *villin* promoter develop colonic polyposis including dysplastic serrated polyps, but no mucinous adenocarcinoma. *Smad3*^*-/-*^ mice, deficient in the transforming growth factor beta (TGFβ) signaling molecule SMAD3, develop inflammation-associated dysplasia and mucinous colon cancer, but only after chemically induced epithelial injury [41]. Additionally, loss of Notch activation, and subsequent down-regulation of Hes-1 was shown to lead to colitis and adenocarcinoma, in a fucosylation deficient (*Fx*^*-/-*^) mouse model [42]. Interestingly, lesions resembling human sessile serrated adenomas arise during colitis in *Fx*^*-/-*^ mice and these mice further develop mucinous adenocarcinomas. While the *VN*^*-/-*^ mice do not develop colitis, the histological features of sessile serrated adenoma like lesions are shared with the *Fx*^*-/-*^ model. Both models also have low-grade dysplasia that evolves into high-grade dysplasia over time, due to the loss of Notch-1 [42]. Thus, we and others show the potentially protective role of Notch-1, and in turn Hes-1, in murine models of mucinous CRC.

Most studies on human CRC demonstrate that Notch-1 is elevated in human CRC tissues and correlated with poor differentiation, tumor progression and poor survival [30, 43–46]. Notch-1, Jagged-1 and Jagged-2 (two of the 5 Notch ligands), and its target gene Hes-1 are expressed in human primary adenocarcinomas and colorectal cancer cell lines [27–30]. Additionally, enhanced Notch-1 signaling in colorectal cancer is simultaneously linked to the ability of Wnt/β-catenin to activate Notch-1 ligands, which appear to be critical for the growth of colorectal adenomas [6, 28, 29, 47]. Recent data also demonstrates that inhibition of Notch-1/Jagged1 by alteration of microRNA miR-34a prevents tumor cell invasion and metastasis in CRC cell lines [48]. Similar to mouse data using APC^Min^ mice, Notch was found to act as a mediator in intestinal tumorigenesis in familial adenomatous polyposis [29]. Conversely, a few studies have reported decreased Hes-1 levels in colon carcinomas compared with adenomas, indicating that the involvement of Notch-1 signaling in the initiation and development of CRC remains unclear and controversial [6, 30, 49]. This is further corroborated by the finding that right-sided CRC shows complete loss of Hes-1 [42].

One reason for this discrepancy in providing clear and conclusive evidence for the role of Notch-1 in CRC is the lack of studies using patient stratification based on CRC subtypes. This is validated by the description of high, low and no-Notch-1 CRC expressers, in humans [43]. Additionally, decreased expression of Wnt target genes via a tumor suppressive effect of Notch-1 has been described in human CRC [32]. Our analysis demonstrates that the expression of Notch-1 is significantly reduced in human colorectal mucinous adenocarcinoma when compared to non-mucinous colorectal adenocarcinoma. Although Atoh-1 has been shown to function as a tumor suppressor [50–52], patients with mucinous colorectal tumors exhibit high levels of Atoh-1 expression and retain prominent secretory cell components possibly due to the absence or inactivation of Notch-1 signaling [53, 54]. Altogether, our data underscore critical role of Notch-1 in the intestinal epithelial compartment during mucinous CRC, highlighting the pivotal role of the epithelium in this pathological context.

CRC can be classified into several subtypes with distinct molecular and genomic signatures that will translate into personalized therapies. Our efforts have identified a new molecular signature for the subset of mucinous CRC in humans. Mucinous CRC patients would largely benefit from a more targeted approach of screening, diagnosis and treatments toward the Notch-1 signaling pathway. In summary, our findings reveal a novel role for epithelial Notch-1 in protecting from mucinous colorectal adenocarcinoma and open avenues for the development of personalized medicine and targeted therapeutics to be tailored to specific types of colon cancers.

## MATERIALS AND METHODS

### Mice

Wild-type (WT; C57BL/6J), Notch-1 flox/flox (*Notch-1*^*fl/fl*^) and *Villin*^*Cre*^ mice were purchased from Jackson Laboratories and housed in the Icahn School of Medicine at Mount Sinai specific pathogen-free barrier facility. Notch-1 flox/flox (*Notch-1*^*fl/fl*^) mice are known to have a normal phenotype with no changes in Notch-1 expression [55]. *Villin-Cre/Notch-1*^*fl/fl*^ (*VN*^*-/-*^) mouse strain was generated by crossing *Villin*^*Cre*^ and *Notch-1*^*fl/fl*^ mice. All mice were genotyped according to Jackson Laboratories’ protocols at the Transgenix. Mice were used at 10 or 30 weeks of age. Experiments were carried out using age and gender matched groups. The Institutional Animal Care and Use Committee approved all procedures performed in this study.

### Ussing chamber

Permeability studies were performed on colonic tissues from WT and *VN*^*-/-*^ mice using FITC-Dextran (Sigma-Aldrich, St. Louis, MO, USA) in Ussing chambers (Physiological Instruments, San Diego, CA, USA) as previously described [17]. Briefly, fresh tissue was mounted on a chamber slide connected to voltage-clamp Ussing chambers. A 10mmol/L glucose-Kerbs solution was added to the serosal side, and a 10mmol/L mannitol-Krebs solution was added to the luminal side. Electrophysiological measurements were performed using a transepithelial voltage clamp. The resistance was measured after 30 minutes. FITC-Dextran was added to the luminal side and the permeability of the tissue was assessed by examining flux of fluorescence across the mucosa to the serosal compartment every 30 minutes for 2 hours [17].

### Quantitative real-time PCR

RNA was extracted from homogenized colonic tissues using Trizol (Life technologies). RNA was reverse transcribed using the qScript cDNA system (Quanta Biosciences, Gaithersburg, MD, USA). *Notch-1* and *Actin* mRNA expression was evaluated using the Solaris qPCR Gene Expression Assay system accompanied by the Solaris probes (Thermo Scientific). For all the other genes, mRNA expression was quantified using the SYBR Advantage qPCR system (Clontech Laboratories, Mountain View, CA, USA) (Supplementary Table 1 for primer list and sequences). Experiments were performed and analyzed using the Applied Biosystems VIIA7 Real-Time PCR platform (Life Technologies). Transcripts were assayed in duplicate, qPCR results were normalized to *L32* or *Actin* expression and relative expressions calculated using the ΔΔCT method.

### Immunofluorescence and immunohistochemistry

Paraffin-embedment and hematoxylin and eosin (H&E) stains were performed at the Histology core facility at the Icahn School of Medicine at Mount Sinai. Histologic changes were evaluated and scored by a pathologist blinded to the treatment group. For immunofluorescence, de-waxed paraffin-embedded colonic tissue sections were stained as previously described [17, 19]. Briefly, tissue sections were incubated with primary antibodies against MUC2 (SantaCruz), and pan-keratin (Abcam) followed by the appropriate secondary antibodies (Life Technologies), and counterstained using 4’, 6’-diamidino-2-phenylindole (DAPI). All slides were examined on a Nikon Eclipse Ni series microscope and images captured at 4x, 10x and 20x magnifications.

### Oncomine database analysis

Gene expression data of *Notch-1* was obtained from three colorectal (CRC) datasets using the Oncomine database as previously described [56] (https://www.oncomine.com/). Fold change of *Notch-1* mRNA expression acquired from each CRC dataset was compared between CRC and mucinous CRC tissues using the following filters: threshold fold change ≥ 1.5X; *p* value ≥ 1E-04; and gene rank in the top 10%. Expression values of *Notch-1* were sorted based on *p* values and presented in Log2 median-centered intensity ratios for three CRC datasets including: TCGA Colorectal [57]; Bittner Colon; and Kaiser Colon [58].

### Statistical analysis

Statistical significance was determined using two-tailed Mann Whitney test (2 groups comparisons only). A value of *p<0.05* was considered significant. All analyses were performed and data was graphed with Prism software (GraphPad, La Jolla, CA, USA).

## SUPPLEMENTARY MATERIALS TABLE



## Abbreviations

Atoh-1: (Atonal homolog-1)
CRC: (colorectal cancer)
RBP-J: (Recombinant Binding Protein Suppressor of Hairless)
Hes-1: (Hairy and enhancer of split-1)
VN^*-/-*^: *(Villin-Cre/Notch-1*^*fl/f*^*)*
