# Supplementary material for: Intestinal epithelial Notch-1 protects from colorectal mucinous adenocarcinoma
Source: Oncotarget. 2018 Sep 11;9(71):33536–48. doi: 10.18632/oncotarget.26086 (PMC6173356; doi:10.18632/oncotarget.26086)
Supplement: Supplementary file 1 [file oncotarget-09-33536-s001.pdf]

Intestinal epithelial Notch-1 protects from colorectal mucinous adenocarcinoma

SUPPLEMENTARY MATERIALS

Supplementary Table 1: Design of the rtPCR murine primers

Murine Gene	Primer Sequences
Cdc2	fwd 5'-TCGCATCCCACGTCAAGA-3'
	rev 5'-GTTTGGCAGGATCATAGACTAGCA-3'
c-Myc2	fwd 5'-TCTCCACTCACCAGCACAACTACG-3'
	rev 5'-ATCTGCTTCAGGACCCT-3'
Cyclin B1	fwd 5'-ACTTCAGCCTGGGTCGCC-3'
	rev 5'-ACGTCAACCTCTCCGACTTTAGA-3'
Cyclin E	fwd 5'-ATGTGGCCGTGTTTTGCA-3'
	rev 5'-GGTCTGATTTTCCGAGGCTGA-3'
Angiogenin-4	fwd 5'-TCCAGGAGCACACAGCTAGA-3'
	rev 5'-GCTTGGCATCATAGTGCTGA-3'
Cox2	fwd 5'-TGAGCACAGGATTTGACCAG-3'
	rev 5'-CCTTGAAGTGGGTCAGGATG-3'
Hif1α	fwd 5'-TGCTCATCAGTTGCCACTTC-3'
	rev 5'-CCATCTGTGCCTTCATCTCA-3'
MMP10	fwd 5'-CACAAGCCCAGCTAACTTCC-3'
	rev 5'-TTTGTCTGGGGTCTCAGGTC-3'
Amphiregulin	fwd 5'-CATCGGCATCGTTATCACAG-3'
	rev 5'-ACAGTCCCGTTTTCTTGTCG-3'
Epiregulin	fwd 5'-CACCGAGAAAGAAGGATGGA-3'
	rev 5'-GATTCTCCTGGGATGCATGA-3'
Wnt5a	fwd 5'-GGTGCCATGTCTTCCAAGTT-3'
	rev 5'-TGAGAAAGTCCTGCCAGTTG-3'
